# Supplementary material for: Content-rich biological network constructed by mining PubMed abstracts
Source: BMC Bioinformatics. 2004 Oct 8;5:147. doi: 10.1186/1471-2105-5-147 (PMC528731; doi:10.1186/1471-2105-5-147)
Supplement: Additional File 2 — The original results of the above study (non-essential files are deleted to keep the file size under the limit set by BMC bioinformatics). [file 1471-2105-5-147-S2.bz2 › chilibotAdditionalFile2/dip05/264ID7597098E1063/html/CDC42_PAK1.html]

 


 **CDC42** and **PAK1** 
  
Found 308 abstracts in PubMed, retrieved 05.  
 

 What does Google say? 
 PDF only 
| .edu only 

---

**Interactive relationship** (e.g. stimulation, inhibition, etc)

**Neutral relationship**- Our results suggest that Gbetagamma binds  **PAK1**  and, via PAK  [ **PAK1** ]  associated PIXalpha, activates  **Cdc42** , which in turn activates  **PAK1** .  Ref: 12887923 Cell, 2003
- p21  [ **PAK1** ]  activated protein kinases PAK  [ **PAK1** ]  are a family of serine threonine protein kinases which are activated by binding of the p21  [ **PAK1** ]  G proteins  **Cdc42**  or Rac.  Ref: 12853446 J Biol Chem< MedlineTA>J Biol Chem, 2003
- Two reports in this issue show that activated  **Cdc42**  at the leading edge helps orient the cell s axis in a signaling complex with Gbetagamma,  **PAK1** , and PIXalpha.  Ref: 12887916 Cell, 2003
- The p21  [ **PAK1** ]  activated protein kinases Paks are serine threonine protein kinases activated by binding to Rho family small GTPases, Rac and  **Cdc42** .  Ref: 12860998 J Biol ChemJ Biol Chem, 2003

**Non-interactive relationship** (e.g. studied together, co-existance, homology, etc.)

- Mutation of histidine residues 19 and 22 to leucine on the p21  [ **PAK1** ]  binding domain of Pak5 completely abolished the binding of  **Cdc42**  and the  **Cdc42**  mediated autophosphorylation.  Ref: 12860998 J Biol ChemJ Biol Chem, 2003
- Taken together, these results suggest that Pak5, like  **Pak1** , contains an autoinhibitory domain and its activity is regulated by  **Cdc42** .  Ref: 12860998 J Biol ChemJ Biol Chem, 2003
- This Gbetagamma  **PAK1**  PIXalpha  **Cdc42**  pathway is essential for the localization of F actin formation to the leading edge, the exclusion of PTEN from the leading edge, directional sensing, and the persistent directional migration of chemotactic leukocytes.  Ref: 12887923 Cell, 2003
- Directional Sensing Requires Gbetagamma Mediated  **PAK1**  and PIXalpha Dependent Activation of  **Cdc42** .  Ref: 12887923 Cell, 2003
- We describe a signaling mechanism involving Gbetagamma, PAK  [ **PAK1** ]  associated guanine nucleotide exchange factor PIXalpha,  **Cdc42** , and p21  [ **PAK1** ]  activated kinase PAK 1  [ **PAK1** ] .  Ref: 12887923 Cell, 2003
- Thus, in this pathway,  **PAK1**  is not only an effector for  **Cdc42** , but it also functions as a scaffold protein required for  **Cdc42**  activation.  Ref: 12887923 Cell, 2003
